# Supplementary material for: Modeling glioblastoma heterogeneity as a dynamic network of cell states
Source: Mol Syst Biol. 2021 Sep 16;17(9):e10105. doi: 10.15252/msb.202010105 (PMC8444284; doi:10.15252/msb.202010105)
Supplement: Supplementary file 6 — Source Data for Figure 5 [file MSB-17-e10105-s004.zip › Figure5A_sourcedata/GSEA_3071/hallmarks_stateIII.GseaPreranked.1623742540963/HALLMARK_ADIPOGENESIS.html]

Details for gene set HALLMARK\_ADIPOGENESIS[GSEA]

|  || Dataset | state33071 |
| Phenotype | NoPhenotypeAvailable |
| Upregulated in class | na\_neg |
| GeneSet | HALLMARK\_ADIPOGENESIS |
| Enrichment Score (ES) | -0.23294836 |
| Normalized Enrichment Score (NES) | -1.2803367 |
| Nominal p-value | 0.20762712 |
| FDR q-value | 0.3662535 |
| FWER p-Value | 0.999 |
Table: GSEA Results Summary

  

Fig 1: Enrichment plot: HALLMARK\_ADIPOGENESIS      
 Profile of the Running ES Score & Positions of GeneSet Members on the Rank Ordered List

  

| PROBE | GENE SYMBOL | GENE\_TITLE | RANK IN GENE LIST | RANK METRIC SCORE | RUNNING ES | CORE ENRICHMENT || 1 | UQCRQ |  |  | 13 | 0.574 | 0.0219 | No |
| 2 | COX8A |  |  | 63 | 0.448 | 0.0290 | No |
| 3 | UQCR10 |  |  | 89 | 0.421 | 0.0411 | No |
| 4 | COX7B |  |  | 135 | 0.386 | 0.0464 | No |
| 5 | COX6A1 |  |  | 145 | 0.379 | 0.0608 | No |
| 6 | UQCR11 |  |  | 225 | 0.326 | 0.0548 | No |
| 7 | DDT |  |  | 262 | 0.304 | 0.0589 | No |
| 8 | SOD1 |  |  | 315 | 0.287 | 0.0581 | No |
| 9 | SDHB |  |  | 323 | 0.286 | 0.0688 | No |
| 10 | ATP5PO |  |  | 337 | 0.282 | 0.0779 | No |
| 11 | NDUFA5 |  |  | 346 | 0.278 | 0.0881 | No |
| 12 | MDH2 |  |  | 393 | 0.262 | 0.0877 | No |
| 13 | ALDOA |  |  | 440 | -0.251 | 0.0869 | No |
| 14 | DNAJB9 |  |  | 448 | -0.251 | 0.0961 | No |
| 15 | MYLK |  |  | 479 | -0.253 | 0.0996 | No |
| 16 | MIGA2 |  |  | 550 | -0.257 | 0.0928 | No |
| 17 | GPHN |  |  | 625 | -0.261 | 0.0852 | No |
| 18 | CHUK |  |  | 644 | -0.262 | 0.0921 | No |
| 19 | AGPAT3 |  |  | 665 | -0.263 | 0.0985 | No |
| 20 | LPCAT3 |  |  | 682 | -0.264 | 0.1059 | No |
| 21 | ATP1B3 |  |  | 937 | -0.276 | 0.0525 | No |
| 22 | FZD4 |  |  | 1058 | -0.282 | 0.0340 | No |
| 23 | ANGPT1 |  |  | 1184 | -0.289 | 0.0145 | No |
| 24 | ELOVL6 |  |  | 1332 | -0.296 | -0.0104 | No |
| 25 | PEX14 |  |  | 1415 | -0.301 | -0.0184 | No |
| 26 | PFKFB3 |  |  | 1519 | -0.307 | -0.0314 | No |
| 27 | SLC27A1 |  |  | 1555 | -0.310 | -0.0268 | No |
| 28 | TOB1 |  |  | 1573 | -0.310 | -0.0175 | No |
| 29 | GPX3 |  |  | 1638 | -0.315 | -0.0202 | No |
| 30 | SORBS1 |  |  | 1691 | -0.318 | -0.0196 | No |
| 31 | ADCY6 |  |  | 1982 | -0.334 | -0.0798 | No |
| 32 | PHLDB1 |  |  | 2001 | -0.335 | -0.0697 | No |
| 33 | FAH |  |  | 2007 | -0.335 | -0.0562 | No |
| 34 | GPAM |  |  | 2154 | -0.345 | -0.0787 | No |
| 35 | SLC19A1 |  |  | 2367 | -0.363 | -0.1174 | No |
| 36 | DHCR7 |  |  | 2654 | -0.386 | -0.1742 | No |
| 37 | SOWAHC |  |  | 2686 | -0.389 | -0.1651 | No |
| 38 | DGAT1 |  |  | 2693 | -0.390 | -0.1495 | No |
| 39 | MCCC1 |  |  | 2944 | -0.414 | -0.1958 | No |
| 40 | SCARB1 |  |  | 2975 | -0.417 | -0.1852 | No |
| 41 | NKIRAS1 |  |  | 3161 | -0.439 | -0.2136 | Yes |
| 42 | SLC5A6 |  |  | 3204 | -0.443 | -0.2050 | Yes |
| 43 | RETSAT |  |  | 3245 | -0.450 | -0.1955 | Yes |
| 44 | COL15A1 |  |  | 3317 | -0.464 | -0.1934 | Yes |
| 45 | NABP1 |  |  | 3405 | -0.481 | -0.1946 | Yes |
| 46 | ABCA1 |  |  | 3421 | -0.485 | -0.1772 | Yes |
| 47 | OMD |  |  | 3481 | -0.501 | -0.1703 | Yes |
| 48 | LAMA4 |  |  | 3587 | -0.542 | -0.1736 | Yes |
| 49 | ITIH5 |  |  | 3619 | -0.560 | -0.1569 | Yes |
| 50 | APLP2 |  |  | 3693 | -0.610 | -0.1489 | Yes |
| 51 | ENPP2 |  |  | 3713 | -0.627 | -0.1262 | Yes |
| 52 | ITGA7 |  |  | 3730 | -0.639 | -0.1022 | Yes |
| 53 | SSPN |  |  | 3808 | -0.756 | -0.0888 | Yes |
| 54 | COL4A1 |  |  | 3829 | -0.791 | -0.0591 | Yes |
| 55 | CAVIN2 |  |  | 3860 | -0.862 | -0.0289 | Yes |
| 56 | APOE |  |  | 3891 | -1.056 | 0.0098 | Yes |
Table: GSEA details [plain text format]

  

Fig 2: HALLMARK\_ADIPOGENESIS: Random ES distribution      
 Gene set null distribution of ES for **HALLMARK\_ADIPOGENESIS**
